# Impact of the national centralized volume-based procurement policy on antihypertensive drug procurement, price, and volume in Guangxi: an interrupted time series analysis of procurement data

**DOI:** 10.3389/fphar.2026.1810714

**Published:** 2026-06-29

**Authors:** Shuang Liang, Shuang Zhang, Ling Li, Jing Luo, Lang Jiang, Jin Xian

**Affiliations:** 1 Guangxi University of Chinese Medicine, School of Public Health and Management, Nanning, Guangxi, China; 2 Guangxi Center for Food and Drug Evaluation and Inspection, Nanning, Guangxi, China

**Keywords:** drug procurement, hypertension, interrupted time series, volume-based procurement, western China

## Abstract

**Introduction:**

The impact of China’s national centralized volume-based procurement (NVBP) policy on antihypertensive drug procurement in underdeveloped, multi-ethnic regions remains understudied. This study evaluated changes in procurement prices, volumes, and costs for four antihypertensive drugs following the second NVBP batch in Guangxi, an economically disadvantaged region in southwestern China.

**Methods:**

Monthly procurement data for four NVBP antihypertensive drugs (Olmesartan Medoxomil, Candesartan Cilexetil, Terazosin Hydrochloride, and Indapamide) were collected from January 2019 to April 2021, covering 16 months before and 12 months after policy implementation (May 1, 2020). Interrupted time series (ITS) analysis with Newey-West standard errors was used to assess immediate and sustained changes in log-transformed defined daily doses (DDDs) and defined daily dose costs (DDDc). Sensitivity analyses examined robustness to a January 2021 procurement spike.

**Results:**

Following policy implementation, the average unit price of the four drugs decreased by 81.52%, and median DDDc dropped by 96.88% to 99.78%. ITS models showed significant immediate increases in DDDs for all four drugs (all *p* < 0.001), but no significant sustained long-term trend (all *β*_*3*_ not significant, *p* > 0.05). A sharp DDDs spike occurred in January 2021, followed by a decline. Sensitivity analyses confirmed that the spike did not affect the direction or significance of the main estimates.

**Discussion:**

The second NVBP batch was associated with substantial and sustained reductions in procurement prices and costs for these four antihypertensive drugs in Guangxi. However, procurement volumes showed marked volatility without stable long-term growth. These findings, based on procurement records rather than patient-level data, suggest that price reductions alone may not ensure consistent procurement patterns. Strengthening supply chain resilience and aligning procurement schedules with clinical demand may support more stable drug supply in disadvantaged regions.

## 1 Introduction

The rise in medical costs is a worldwide challenge, and due to incomplete competition in the pharmaceutical market, government regulation is necessary in various countries (Marquis and Phelps, 1987). Centralized volume-based procurement of drugs is an internationally common purchasing method to reduce patients’ medication burden (Seidman and Atun, 2017). In low-income and middle-income countries (LMICs), pooled procurement has been shown to lower prices, though implementation challenges remain (Seidman and Atun, 2017). Existing international evidence has primarily focused on price reduction effects at the national or regional level, with documented average savings ranging from 15% to over 50% depending on the context (Wang and Zahur, 2025; Dubois et al., 2019). However, post-policy utilization patterns—particularly whether price reductions translate into sustained and stable medication use—have received less attention. Furthermore, most studies have been conducted in relatively centralized or high-income settings, leaving limited empirical evidence on the implementation of such policies in economically disadvantaged, multi-ethnic regions where healthcare infrastructure is weaker and chronic disease burdens are high (Costa et al., 2026). Since 2019, China has conducted ten batches of nationally organized centralized volume-based drug procurement to squeeze out inflated drug prices and reduce patients’ treatment burden, accumulating a total of 435 drug varieties procured with an average price reduction of over 50%. Researchers from various fields pay great attention to the effects brought about by the centralized drug procurement policy.

Regional economies of scale suggest that national centralized drug procurement can influence the quantity and cost of medicines (Wang J. et al., 2024). Prior studies indicate that the implementation of the centralized drug procurement policy has been associated with reduced drug prices and changes in procurement volumes in China (Zhao et al., 2025). Pharmaceutical companies, considering the economic leverage between market share and unit net profit during bidding, may proactively reduce drug prices (Wang J. et al., 2024).

The impact of the centralized procurement policy varies across different regions and diseases. In China, medical resources often exhibit a characteristic of being more abundant in the eastern regions than in the western regions (Chai et al., 2020). Most evaluations have been conducted in central and eastern developed areas, while rigorous evidence from underdeveloped, multi-ethnic regions in southwestern China remains scarce.

Geographic variation in healthcare utilization and expenditure is well documented in China, with substantial disparities driven by a mix of demand- and supply-side factors across regions. Cao and Pan (Cao and Pan, 2024) provided a rigorous methodological framework for analyzing such geographic heterogeneity using small-area analysis, showing that demand factors and supply factors together explain most regional variation in health spending. Their work underscores the importance of regional-level analysis rather than relying on national averages, especially in economically disadvantaged regions with weaker health systems.

Guangxi Zhuang Autonomous Region is an underdeveloped area in southwestern China with a large population and a high prevalence of hypertension. Hypertension is highly prevalent in Guangxi, with reported rates above 36%, considerably higher than the national average (He et al., 2023; Tang et al., 2023). Long-term pharmacotherapy is essential for hypertension management, creating a substantial economic burden for patients. However, *per capita* healthcare expenditure in Guangxi is below the national average, and local healthcare capacity is relatively limited.

Guangxi has fully implemented the second batch of national NVBP, yet no interrupted time series (ITS) study has evaluated short-term changes in procurement-related indicators for antihypertensive drugs. In particular, it remains unclear whether price reductions are accompanied by stable procurement trends or high volatility.

This paper takes the antihypertensive drugs from the second batch of nationally organized centralized volume-based procurement (hereinafter referred to as “centralized procurement”) as the research subject. By analyzing procurement price and quantity indicators, the aim is to provide a reference for strengthening Guangxi’s capacity to formulate centralized drug procurement policies, to contribute to the healthy development of Guangxi’s healthcare sector, and to offer some preliminary insights for global health policy, particularly in the context of procurement strategies and their implications for procurement-based drug utilization in low-resource, multi-ethnic settings.

## 2 Methods

### 2.1 Data sources

The procurement data were sourced from two official databases in Guangxi Zhuang Autonomous Region: (i) the Guangxi Public Resource Trading Platform, from which pre-policy transaction records (January 2019 - April 2020) were extracted, and (ii) the Guangxi Medical Security Bureau, from which pos-policy transaction records (May 2020 - April 2021) were extracted. Both databases contain the same underlying procurement transaction records; the datasets were cross-checked for consistency. DDD values (mg) were obtained from the WHO ATC/DDD Index (2025 version).

#### 2.1.1 Data acquisition and cleaning

Data were extracted in 2025. Two raw datasets were received:

Pre-policy data - 16 monthly worksheets, each with one row per drug, containing unit price (CNY), total procurement amount (CNY), and total quantity (tablets/capsules).

Post-policy data - 12 monthly worksheets in the same structure, with an additional column for dose specification (e.g., 1 mg, 2 mg) because several drugs had multiple winning products of different strengths.

This study used Microsoft Excel 2019 for processing raw data and descriptive analysis. File consolidation. All monthly worksheets were merged. A dose_per_tab column was added to the pre-policy dataset to align with the post-policy structure. The merged table contained columns: date (YYYY-MM-DD), product (generic name), volume (total expenditure, CNY), amount (total quantity, tablets/capsules), ddd (mg), and dose per tab.

Product name standardization. All brand-name products were collapsed to the official generic name.

Duplicate and missing record removal. Duplicate records and records with missing price or quantity were removed.

Monthly aggregation. Volume and amount were summed by drug, dose, and calendar month.

The cleaned dataset is a balanced 28-month panel for each drug. Raw procurement records are available upon reasonable request subject to institutional approval.

### 2.2 Research subjects

To avoid the generality of macro-level evaluations, the second batch of the NVBP policy was selected for three reasons. First, unlike the pilot “4 + 7” batch, the second batch was implemented nationwide with a mature policy design, enhancing generalizability. Second, its complete procurement cycle (1–3 years) provides sufficient data for ITS analysis to distinguish immediate from long-term effects. Third, antihypertensive drugs—the largest category in Guangxi’s second batch—are strategically important: they showed substantial price reductions, their utilization (DDDs) reflects prescribing continuity, and their cost burden is high.

Thus, the antihypertensive drugs from the second batch of centralized procurement in the Guangxi Zhuang Autonomous Region were selected as the research subjects. There were four drugs in the second batch: Olmesartan Medoxomil Tablets, Candesartan Cilexetil Tablets, Terazosin Hydrochloride Capsules, and Indapamide Tablets. Monthly aggregated procurement records for these four centralized procurement antihypertensive drugs, including unit drug price, drug procurement quantity, and total drug procurement amount, were tracked and analyzed.

#### 2.2.1 Time window selection

The observation period from January 2019 to April 2021 was determined by three factors. (i) January 2019 was the earliest month for which complete, verified procurement records were available from both the Guangxi Medical Security Bureau and the Public Resource Trading Platform after a system upgrade of the provincial procurement data repository in late 2018. (ii) To construct a robust interrupted time series design, a minimum of 12 months of pre-intervention data was required to estimate the baseline trend reliably, and at least 12 months of post-intervention data to assess the immediate and short-term trajectory changes. The second NVBP batch was implemented on 1 Ma y 2020, making April 2021 the 12th post-policy month, thus yielding 16 pre-intervention and 12 post-intervention months. (iii) data were extracted in 2025 using the most recent fully audited records, which ran through April 2021.

To assess whether extending the observation window would alter the conclusions, we conducted an exploratory analysis incorporating three additional months (May-July 2021) that had not yet been fully audited at the time of extraction. The direction and statistical significance of the immediate level change (*β*_*2*_) for both DDDs and DDDc remained unchanged in this extended dataset, indicating that the primary findings are not sensitive to the chosen cut-off date. However, because these additional months were not fully verified, they were excluded from the main analysis.

### 2.3 Research methods

#### 2.3.1 Defined daily dose system (DDDs) and defined daily dose cost (DDDc)

The Defined Daily Dose Cost analysis was used to calculate the total procurement amount, DDDs, and DDDc of the antihypertensive drugs. The WHO-ATC/DDD system was used. For each drug, the DDD (mg) is the assumed average maintenance dose per day for its main indication in adults. Two indicators were calculated:

Total Defined Daily Doses (DDDs):

$$\text{DDDs}=\frac{\text{Total}\;\text{procured}\;\text{amount}\;\left(\text{mg}\right)}{\text{DDD}\left(\text{mg}\right)}$$

DDDc represents the acquisition cost per standard daily dose.

Defined Daily Dose Cost (DDDc):

$$\text{DDDc}\left(\text{CNY}/\text{DDD}\right)=\frac{\text{Total}\;\text{procurement}\;\text{expenditure}\;\left(\text{CNY}\right)}{\text{DDDs}}$$

#### 2.3.2 Interrupted time series (ITS)

To assess changes associated with the centralized procurement policy on procurement indicators of antihypertensive drugs and reveal the dynamic pattern of the intervention effect, the study employed Interrupted Time Series analysis. Monthly data such as DDDs and DDDc for the four antihypertensive drugs included in the second batch of centralized procurement before and after policy implementation were tracked and analyzed. The policy intervention point was May 2020, when the Guangxi Medical Security Bureau issued the “Notice on Implementing the Second Batch of Nationally Organized Drug Centralized Procurement and Use Work”. The period from January 2019 to April 2020 (16 months) was defined as pre-policy implementation, and the period from May 2020 to April 2021 (12 months) was defined as post-policy implementation.

Monthly defined daily doses (DDDs) and defined daily dose cost (DDDc) were natural-log transformed before analysis to stabilise variance and improve residual normality. For each outcome, we fitted the following segmented regression model:

$$\ln\left(Y_{t}\right)=\beta_{0}+\beta_{1}X_{1}+\beta_{2}X_{2}+\beta_{3}X_{3}+\varepsilon$$

Y_t_ is the average level of the above dependent variable at time point t (month); X_1_ is a continuous time variable measured in months during the observation period, ranging from 1 to 28, corresponding sequentially to the observation time points; X_2_ is a dummy categorical variable, taking the value 0 before policy intervention and 1 after policy intervention; X_3_ is the time variable from policy implementation to the end of the study, ranging from 0 to 12; *β*_*0*_ is the estimated initial level of the dependent variable (t = 1); *β*_*1*_ is the trend of the dependent variable over time before the intervention, i.e., the slope of the monthly DDDc and DDDs trend for antihypertensive drugs before the centralized procurement policy implementation; *β*_*2*_ is the instantaneous change at the time of intervention, reflecting the immediate effect of policy implementation; *β*_*3*_ is the change in slope before and after the intervention, reflecting the sustained effect of the policy implementation.

##### 2.3.2.1 Seasonal adjustment

For each outcome-drug combination, we tested whether adding month-of-year dummy variables (January-November, with December as reference) significantly improved the model fit using an F-test. If the joint test was significant at α = 0.10, the final model retained the monthly dummies; otherwise the more parsimonious specification was used. Seasonality adjustments were applied only to the DDDs models for Indapamide and Terazosin (season *p* = 0.007 and *p* = 0.003, respectively). All DDDc models and the remaining DDDs models used the base specification.

##### 2.3.2.2 Robust inference

Models were estimated by ordinary least squares (OLS). Residual autocorrelation was assessed with the Ljung-Box test (lag = 6) and heteroscedasticity with the Breusch-Pagan test; residual normality was checked with the Shapiro-Wilk test. Because mild autocorrelation and/or heteroscedasticity were detected in several models, we employed three robust approaches (Marquis and Phelps, 1987): Newey-West heteroscedasticity- and autocorrelation-consistent (HAC) standard errors with a fixed lag of 4 months (Seidman and Atun, 2017); Prais-Winsten generalised least squares assuming AR (1) errors; and (Wang and Zahur, 2025) percentile bootstrap 95% confidence intervals.

##### 2.3.2.3 Log-transformation decision

Raw DDDs were right-skewed with heteroscedasticity (variance increased with mean). Raw DDDc also deviated from normality due to extremely low values in the post-intervention period. To stabilise variance and improve normality, both DDDs and DDDc were natural-log transformed before model fitting. After transformation, Shapiro-Wilk tests indicated that the residuals of all DDDs models satisfied the normality assumption (p > 0.05; Supplementary Table S1). For DDDc models, mild non-normality remained for some drugs (Supplementary Table S2), but we relied on Newey-West standard errors and bootstrap confidence intervals, which are robust to such violations.

##### 2.3.2.4 Pulse analysis for January 2021

A sharp spike in DDDs was observed in January 2021. To assess its influence, we re-estimated each DDDs model after adding a pulse dummy variable (1 for January 2021, 0 otherwise). The intervention coefficients (β2 and β3) remained virtually unchanged in direction and significance, confirming the robustness of the main findings. The pulse coefficient itself was significant for Candesartan, Indapamide, and Terazosin, suggesting a temporary volume surge that did not affect the overall policy effect.

### 2.4 Statistical analysis

This study used Microsoft Excel 2019 for processing raw data and descriptive analysis. Difference analysis was performed using SPSS 25.0 software; for non-normally distributed data, the median (M) was used for representation, and the Mann-Whitney U test was used for between-group comparisons, The Mann-Whitney U test was used only for descriptive comparison of medians; it does not account for time trends and is not used for causal inference. Interrupted Time Series analysis and the Autocorrelation Function were performed using RStudio software (version 2022.02.3 + 492, R language environment version 4.4.3). The statistical significance level was set at α = 0.05.

## 3 Results

### 3.1 Basic procurement situation

**FIGURE 1:**
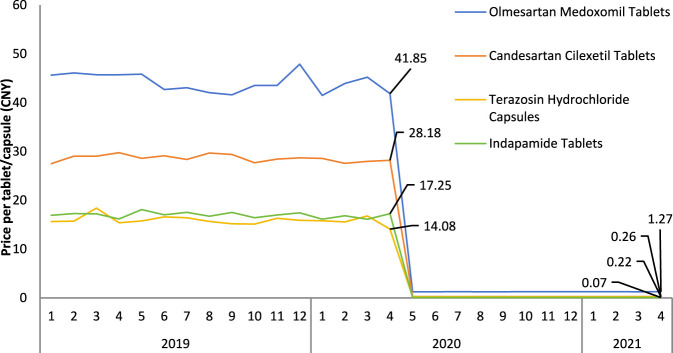
Trend of unit price changes of antihypertensive drugs before and after the NVBP policy.

Regarding the procurement situation before the implementation of Guangxi’s centralized procurement policy, the unit price per tablet/capsule of antihypertensive drugs (Olmesartan Medoxomil Tablets, Candesartan Cilexetil Tablets, Terazosin Hydrochloride Capsules, Indapamide Tablets) varied, but the overall price remained high. After the policy implementation, the unit price of the same specification from the same manufacturer decreased significantly, with an average reduction of 81.52%. The order of price reduction for the four drugs was: Candesartan Cilexetil Tablets (90.16%) > Indapamide Tablets (84.49%) > Olmesartan Medoxomil Tablets (75.99%) > Terazosin Hydrochloride Capsules (75.42%). After centralized procurement, the increase in DDDs for antihypertensive drugs was substantial. The order of DDDs increase was: Indapamide Tablets (2691.00%) > Terazosin Hydrochloride Capsules (1953.11%) > Candesartan Cilexetil Tablets (789.07%) > Olmesartan Medoxomil Tablets (659.27%). Before centralized procurement, the DDDc of antihypertensive drugs was relatively high, all >30 CNY/DDD. After policy implementation, the DDDc decreased dramatically. The DDDc of Indapamide Tablets dropped as low as 0.07 CNY/DDD, with the maximum reduction reaching 99.39%. As shown in Table 1 and Figure 1.

**TABLE 1:** Basic information of the second batch of centrally procured antihypertensive drugs in Guangxi Zhuang autonomous region.

Samples	DDD (mg)	Price (CNY/Tablet/Capsule)		Reduction (%)	DDDs (10,000)		Increase (%)	DDDc (CNY/DDD)		Reduction (%)
		Pre-procurement	Post-procurement		Pre-procurement	Post-procurement		Pre-procurement	Post-procurement	
Olmesartan	20	5.28	1.27	75.99	28.38	215.48	659.27	40.72	1.27	96.88
Candesartan	8	2.68	0.26	90.16	69.18	615.06	789.07	42.51	0.26	99.39
Terazosin	4	1.14	0.28	75.42	11.90	244.32	1953.11	45.95	0.58	98.74
Indapamide	2.5	0.45	0.07	84.49	14.00	390.74	2691.00	31.65	0.07	99.78

### 3.2 Changes in procurement amount, DDDs, and DDDc of antihypertensive drugs before and after policy implementation

After the implementation of the centralized procurement policy, the total procurement amount and DDDc of antihypertensive drugs decreased significantly compared to before, while DDDs increased significantly. The differences were all statistically significant (*p* < 0.01). See Table 2.

**TABLE 2:** Difference analysis of NVBP antihypertensive drugs.

Samples	Total procurement amount			DDDs			DDDc		
	Pre-procurement M	Post-procurement M	*p*	Pre-procurement M	Post-procurement M	*p*	Pre-procurement M	Post-procurement M	*p*
Olmesartan	782,113	236,607	<0.01	18,768	186,515	<0.01	40	1.27	<0.01
Candesartan	2309311	121,801	<0.01	56,443	468,972	<0.01	43	0.26	<0.01
Terazosin	435,523	351,745	<0.01	9630	199,266	<0.01	48	0.58	<0.01
Indapamide	344,377	20,498	<0.01	11,180	308,000	<0.01	32	0.07	<0.01

### 3.3 Analysis results based on the ITS model

Both DDDs and DDDc were natural-log transformed prior to modelling. The final ITS specifications included seasonal adjustment for the DDDs models of Indapamide and Terazosin. Newey-West standard errors (lag = 4) were used as the primary inferential method. Complete model outputs, including OLS coefficients, robust standard errors, bootstrap confidence intervals, and Prais-Winsten estimates, are presented in Tables 3, 4. Residual diagnostics are summarised in Supplementary Tables S1, S2.

**TABLE 3:** Segmented regression estimates for ln (DDDs) with robust inference.

Drug and term	Coefficient (SE)	Newey-west SE (lag 4)	*p* (NW)	Bootstrap 95% CI	Prais-winsten (SE)	*p* (PW)	Adj. *R* ^2^	AIC
Olmesartan	​	​	​	​	​	​	0.937	16.26
Intercept	9.281 (0.153)	0.095	<0.001	8.94, 9.62	9.261 (0.111)	<0.001	​	​
Time	0.070 (0.016)	0.009	<0.001	0.032, 0.107	0.070 (0.012)	<0.001	​	​
Intervention (level)	1.361 (0.228)	0.168	<0.001	0.795, 1.987	1.437 (0.170)	<0.001	​	​
Time after intervention	−0.024 (0.029)	0.021	0.269	−0.097, 0.044	−0.032 (0.021)	0.146	​	​
Candesartan	​	​	​	​	​	​	0.942	13.27
Intercept	10.616 (0.145)	0.089	<0.001	10.23, 10.95	10.592 (0.108)	<0.001	​	​
Time	0.032 (0.015)	0.009	<0.001	−0.0001, 0.066	0.034 (0.011)	0.006	​	​
Intervention (level)	1.705 (0.216)	0.110	<0.001	1.408, 2.114	1.728 (0.165)	<0.001	​	​
Time after intervention	0.008 (0.028)	0.013	0.542	−0.051, 0.063	0.003 (0.020)	0.867	​	​
Terazosin^†^	​	​	​	​	​	​	0.992	−22.36
Intercept	9.498 (0.100)	0.090	<0.001	8.80, 9.39	9.051 (0.067)	<0.001	​	​
Time	0.029 (0.008)	0.008	0.002	−0.001, 0.054	0.031 (0.007)	<0.001	​	​
Intervention (level)	2.506 (0.137)	0.080	<0.001	2.168, 2.851	2.439 (0.103)	<0.001	​	​
Time after intervention	−0.007 (0.016)	0.012	0.772	−0.053, 0.055	−0.0003 (0.013)	0.984	​	​
Indapamide^†^	​	​	​	​	​	​	0.990	−8.55
Intercept	9.612 (0.128)	0.124	<0.001	8.69, 9.51	9.054 (0.093)	<0.001	​	​
Time	0.020 (0.010)	0.011	0.087	−0.017, 0.056	0.025 (0.010)	0.016	​	​
Intervention (level)	3.306 (0.175)	0.080	<0.001	2.827, 3.526	3.122 (0.142)	<0.001	​	​
Time after intervention	−0.029 (0.020)	0.014	0.490	−0.061, 0.051	−0.013 (0.018)	0.462	​	​

^†^ Seasonal dummies retained after significant joint F-test.

SE, standard error. Newey-West SE (lag 4) and p-value are heteroscedasticity- and autocorrelation-consistent. Bootstrap 95% CI, from 1999 replications (percentile method). Prais-Winsten estimates assume AR (1) errors. Adj. *R* ^2^ and AIC, from the final OLS, model. Residual diagnostics are provided in Supplementary Table S1.

**TABLE 4:** Segmented regression estimates for ln (DDDc) with robust inference.

Drug and term	Coefficient (SE)	Newey-west SE (lag 4)	*p* (NW)	Bootstrap 95% CI	Prais-winsten (SE)	*p* (PW)	Adj. *R* ^2^	AIC
Olmesartan	​	​	​	​	​	​	0.9999	−147.44
Intercept	3.671 (0.008)	0.009	<0.001	3.64, 3.69	3.674 (0.011)	<0.001	​	​
Time	0.003 (0.001)	0.001	0.004	0.001, 0.006	0.003 (0.001)	0.015	​	​
Intervention (level)	−3.486 (0.012)	0.010	<0.001	−3.51, −3.46	−3.483 (0.014)	<0.001	​	​
Time after intervention	−0.003 (0.002)	0.001	0.006	−0.006, −0.001	−0.003 (0.002)	0.190	​	​
Candesartan	​	​	​	​	​	​	0.9997	−92.50
Intercept	3.757 (0.022)	0.020	<0.001	3.69, 3.81	3.755 (0.018)	<0.001	​	​
Time	−0.001 (0.002)	0.002	0.699	−0.009, 0.006	−0.001 (0.002)	0.719	​	​
Intervention (level)	−5.094 (0.033)	0.017	<0.001	−5.14, −5.01	−5.091 (0.028)	<0.001	​	​
Time after intervention	0.001 (0.004)	0.002	0.653	−0.006, 0.009	0.001 (0.003)	0.883	​	​
Terazosin	​	​	​	​	​	​	0.999	−58.34
Intercept	3.784 (0.040)	0.039	<0.001	3.66, 3.91	3.786 (0.038)	<0.001	​	​
Time	−0.013 (0.004)	0.004	0.005	−0.028, 0.002	−0.013 (0.004)	0.002	​	​
Intervention (level)	−4.112 (0.060)	0.034	<0.001	−4.20, −3.97	−4.107 (0.057)	<0.001	​	​
Time after intervention	0.012 (0.008)	0.004	0.013	−0.001, 0.027	0.012 (0.007)	0.106	​	​
Indapamide	​	​	​	​	​	​	0.9998	−95.70
Intercept	3.255 (0.021)	0.017	<0.001	3.20, 3.30	3.260 (0.016)	<0.001	​	​
Time	−0.006 (0.002)	0.002	0.009	−0.011, −0.001	−0.007 (0.002)	0.001	​	​
Intervention (level)	−5.870 (0.031)	0.020	<0.001	−5.92, −5.82	−5.858 (0.024)	<0.001	​	​
Time after intervention	0.006 (0.004)	0.002	0.012	0.001, 0.011	0.006 (0.003)	0.079	​	​

#### 3.3.1 DDDs model

**FIGURE 2:**
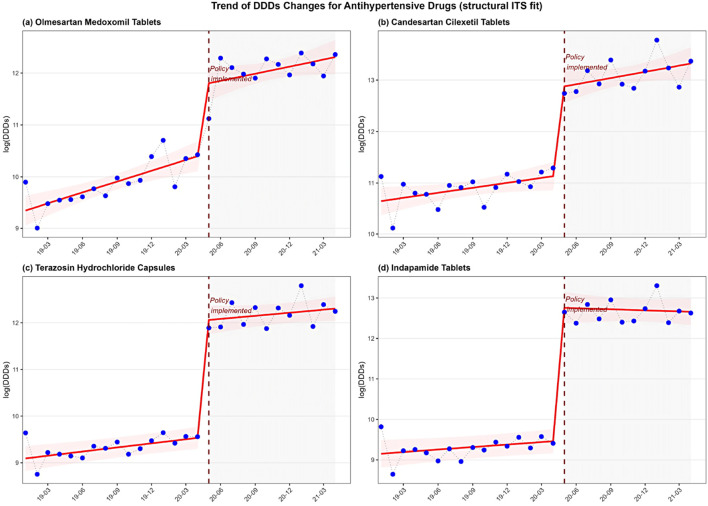
Trend of DDDs changes for antihypertensive drugs. **(a)** Olmesartan Medoxomil Tablets, **(b)** Candesartan Cilexetil Tablets, **(c)** Terazosin Hydrochloride Capsules, **(d)** Indapamide Tablets. The red dashed vertical line indicates the policy implementation date; the shaded grey region marks the post‐intervention period. The solid red line represents the structural ITS fit (linear trend from time, intervention, and time-after only; seasonal effects are controlled but not displayed). The pink translucent band is the 95% confidence interval of the fitted trend. Blue points show observed log-transformed monthly DDDs; grey dotted lines connect consecutive observations.

Table 3 and Figure 2 displays the segmented regression estimates for log-transformed DDDs. After policy implementation, all four drugs showed an immediate and statistically significant increase in procurement volume. The estimated level changes (*β*_*2*_) were positive for every drug, with Newey-West *p* < 0.001 in all cases. The magnitude ranged from 1.36 log-units for Olmesartan (95% bootstrap CI 0.80–1.99) to 3.31 log-units for Indapamide (95% CI 2.827–3.526), corresponding to an approximate 3- to 27-fold increase in DDDs. The pre-intervention trend (*β*_*1*_) was positive and statistically significant for Olmesartan, Candesartan, and Terazosin, but not for Indapamide (Newey-West *p* = 0.087).

The post-intervention trend change (*β*_*3*_) was not statistically significant for any drug (Newey-West *p* > 0.05), suggesting that the sharp rise in DDDs was not followed by a further accelerated or decelerated trend. The Prais-Winsten estimates were consistent with the OLS results, confirming that first-order autocorrelation did not materially affect the conclusions.

The pulse dummy for January 2021 was significant for Candesartan (*β* = 0.66, *p* = 0.022), Indapamide (*β* = 0.71, *p* = 0.012), and Terazosin (*β* = 0.61, *p* = 0.012), but not for Olmesartan (*β* = 0.25, *p* = 0.440). Inclusion of this pulse term had negligible impact on the estimates of *β*_*2*_ and *β*_*3*_, indicating robustness.

Model diagnostics (Supplementary Table S1) showed that the residuals of all four DDDs models did not deviate significantly from normality (Shapiro-Wilk *p* > 0.05). Mild autocorrelation was detected for Indapamide (Ljung-Box *p* = 0.048) and Terazosin (*p* < 0.001), which was corrected by the Newey-West and Prais-Winsten estimators. No heteroscedasticity was found (Breusch-Pagan *p* > 0.05 for all).

#### 3.3.2 DDDc (log-transformed) model

**FIGURE 3:**
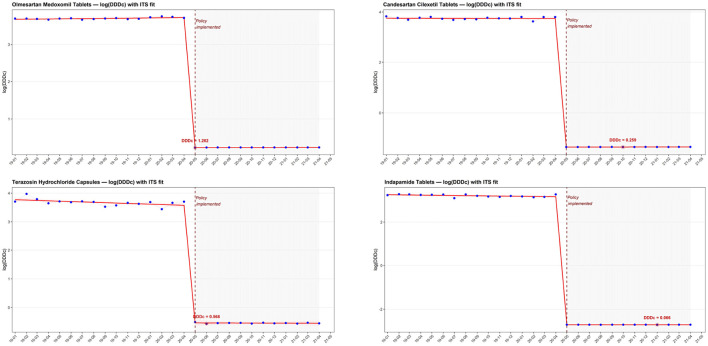
Trend of DDDc changes for antihypertensive drugs.

Table 4 and Figure 3 summarises the ITS estimates for log-transformed DDDc. The intervention level change (*β*_*2*_) was negative and highly significant for all four drugs (Newey-West *p* < 0.001), reflecting an immediate and drastic reduction in procurement cost per DDD. The coefficients ranged from −3.49 (Olmesartan, 95% bootstrap CI -3.51 to −3.46) to −5.87 (Indapamide, 95% CI -5.92 to −5.82), corresponding to a decline of approximately 97%-99.7% in DDDc. The pre-intervention trend (*β*_*1*_) was small and inconsistent across drugs.

The change in trend after the intervention (*β*_*3*_) was significant for Indapamide (*β* = 0.006, Newey-West *p* = 0.012) and Terazosin (*β* = 0.012, *p* = 0.013), indicating a slight upward drift. However, the magnitude of this slope change was minimal compared with the initial level change.

Residual diagnostics (Supplementary Table S2) revealed some deviations from normality (Shapiro-Wilk *p* < 0.05 for Candesartan, Indapamide, and Terazosin) and significant heteroscedasticity (Breusch-Pagan *p* < 0.01 for all drugs). Therefore, inference for DDDc models relies primarily on the Newey-West standard errors and bootstrap confidence intervals, which are robust to these violations. The Prais-Winsten estimates were also fully consistent with the OLS results.

In the DDDc models, the January 2021 pulse was not significant under conventional OLS inference (all *p* > 0.9). After Newey-West correction, the pulse term attained nominal significance for Indapamide (*β* = −0.00094, NW-*p* = 0.005) and Terazosin (*β* = 0.0063, NW-*p* = 0.016), but the associated effect sizes were negligible (<1% change) and did not alter the estimates of *β*_*2*_ or *β*_*3*_. No pulse effect was detected for Candesartan or Olmesartan under any specification.

#### 3.3.3 Sensitivity analysis

Excluding the January 2021 observation from the DDDs models did not change the direction or statistical significance of *β*_*2*_ or *β*_*3*_. Including the spike dummy (reported above) also did not alter the main intervention effect estimates. These checks confirm robustness of the findings to the January 2021 peak.

For DDDc, the pulse effect was materially absent; although Newey-West standard errors indicated a statistically significant but very small pulse for two drugs, the intervention level and slope changes remained unchanged. These checks confirm robustness of the core findings to the January 2021 procurement peak.

## 4 Discussion

### 4.1 Price regulation achieves notable results, while utilization stability requires further attention

The study found that the implementation of the second batch of the national centralized volume-based drug procurement policy in the Guangxi Zhuang Autonomous Region achieved substantial price and DDDc reductions. Following policy implementation, the procurement prices of the four antihypertensive drugs fell sharply, and DDDc decreased immediately and remained low throughout the post-policy period. DDDs also increased immediately. This pattern is consistent with the policy’s core objective of “trading volume for price” and resulted in substantially lower procurement costs for these drugs. While these cost reductions at the procurement level may lead to savings in medication expenses for patients, the lack of patient-level data prevents a direct assessment of this potential benefit, particularly in this underdeveloped western region. However, after the surge, DDDs exhibited high-level volatility and did not form a significant long-term growth trend. This pattern suggests that while the price mechanism functioned effectively in the short term, the translation of lower prices into stable procurement volumes was not consistently achieved for these four drugs.

It is worth noting that the observed average price reduction (81.52%) exceeds the nationally reported average of approximately 50% for the second NVBP batch. Several factors may explain this discrepancy. First, the national average includes all procured drugs across multiple regions, whereas our analysis focuses specifically on antihypertensive drugs in Guangxi, where baseline prices may have been higher due to less competitive markets and higher distribution costs. Second, the official reduction refers to the tender-winning price compared to the previous provincial procurement price, while our calculation reflects actual procurement prices before and after policy implementation, which could capture additional reductions from volume-based discounts. Thus, the larger reduction observed in this study does not contradict the official figure but rather highlights the greater potential for price compression in economically disadvantaged regions.

To further deepen the policy’s effectiveness, the current price reduction achievements should be maintained and the expansion of drug coverage steadily advanced. However, government-led monitoring of procurement stability is warranted to understand whether short-term volume increases can be sustained over longer periods for these specific drugs.

### 4.2 Factors associated with procurement volume fluctuations

Following policy implementation, DDDs exhibited a sharp spike in January 2021, followed by a decline and continued fluctuation. Several potential contributing factors merit consideration, though the present data do not permit empirical testing of these mechanisms.

One potential contributor is seasonal variation in procurement patterns. In China, drug procurement volumes may increase before the spring Festival, possibly reflecting pre-holiday stockpiling by healthcare facilities or patients. The January 2021 peak observed across three of the four drugs coincides with this seasonal window.

A second factor is the COVID-19 pandemic, which emerged in early 2020 and may have influenced drug procurement and supply chains during the study period (Miah et al., 2024; Spieske et al., 2022). In Guangxi, as elsewhere, the pandemic may have altered hospital procurement schedules and led to institutional stockpiling, potentially contributing to the observed volatility. Although ITS analysis controls for underlying time trends, concurrent events such as the pandemic represent an important limitation to causal inference given the absence of a control group.

A third consideration is the potential role of prescriber and patient perceptions. Previous studies have documented that concerns about the quality and efficacy of low-price generic drugs can influence prescribing behavior and patient acceptance (Xing et al., 2022; Wang R. et al., 2024; Huang et al., 2022; Li et al., 2017). If such perceptions existed in Guangxi, they could partly explain why price reductions did not translate into stable procurement growth. Furthermore, procurement data cannot capture substitution effects—whether physicians or patients switched to non-NVBP antihypertensive drugs not included in this analysis. Safety and adverse event profiles of specific antihypertensive agents may also influence prescribing and adherence patterns, and pharmacovigilance studies emphasize the importance of monitoring real-world adverse reactions to inform clinical decisions (Jia et al., 2023). These factors, while plausible, could not be assessed using the aggregated procurement data available.

To mitigate such fluctuations, future policy refinements could consider aligning procurement schedules with anticipated seasonal demand patterns and strengthening supply chain resilience through buffer stock mechanisms and diversified supplier portfolios (Akter et al., 2025). Additionally, communication strategies clarifying the bioequivalence and quality standards of NVBP drugs may help align clinical acceptance with policy goals (Tong et al., 2025; Shen et al., 2025).

### 4.3 Alignment between procured drug varieties and regional therapeutic needs

The release of clinical demand for different centrally procured antihypertensive drugs showed significant divergence: the DDDs increase for Terazosin Hydrochloride Capsules was as high as 1953.11%, while for some varieties (e.g., Olmesartan Medoxomil Tablets) the DDDs increase was relatively lower (659.27%), suggesting differential clinical uptake. This variation may partly reflect differences in baseline utilization, local prescribing preferences, or the therapeutic positioning of these agents within hypertension management.

Whether the four NVBP drugs adequately represent the full spectrum of antihypertensive therapy needs in Guangxi is unclear, as data on non-NVBP antihypertensive utilization were unavailable. The Guangxi experience illustrates the broader challenge of aligning national procurement catalogs with regional disease profiles and prescribing practices. Cao and Pan provided a framework for analyzing geographic heterogeneity in healthcare utilization using small-area analysis, demonstrating that regional demand and supply factors jointly explain substantial variation—an insight that supports the relevance of regionally-informed procurement design (Cao and Pan, 2024). Policymakers could consider establishing a “data-driven + clinical demand” adaptation mechanism: utilizing regional prescribing data to guide the selection of procured varieties and periodically reviewing the catalog alignment with local hypertension treatment patterns. Such an approach would require collaborative efforts between health insurance and health departments.

### 4.4 Interpretation and limitations of the present findings

The findings of this study should be interpreted with several important caveats. First, the ITS design without a control group cannot establish causality; the observed changes represent associations temporally related to policy implementation, and concurrent events (e.g., COVID-19) may have contributed to the patterns observed. Second, the post-policy observation window of 12 months is relatively short, and statements regarding long-term or sustained effects are not supported; longer follow-up is needed to assess whether the policy’s effects persist, attenuate, or evolve. Third, the four drugs analyzed represent a specific subset of second-batch NVBP antihypertensive agents, and findings may not generalize to other drug classes, therapeutic areas, or procurement batches.

The use of aggregated procurement data as a proxy for drug utilization is a fundamental limitation. Procurement records reflect institutional purchasing activity, which may diverge from actual patient consumption due to stockpiling, wastage, or redistribution; they cannot capture patient-level adherence, clinical outcomes, drug exposure, or adverse events. Future research could incorporate pharmacokinetic or therapeutic drug monitoring data to provide more direct measures of actual drug exposure and clinical response, as demonstrated in recent bioanalytical studies (Simultaneous quantification of mirabegron and). Additionally, the absence of patient-level data precluded analysis of whether price reductions translated into reduced financial burden at the individual level, whether out-of-pocket expenditure changed, or whether medication adherence improved. Future studies with patient-level data on expenditures, adherence, and clinical outcomes would substantially strengthen the evidence base.

### 4.5 Future research directions

Building on the limitations identified above, several directions for future research emerge. First, studies incorporating control regions or comparator drugs not subject to NVBP would strengthen causal inference regarding policy effects. Second, longer post-policy observation periods (≥24 months) are needed to assess whether procurement volumes stabilize and whether substitution effects between NVBP and non-NVBP drugs occur. Third, prospective studies collecting patient-level data—including actual medication consumption, clinical outcomes, and patient-reported measures—would bridge the gap between procurement indicators and health impact. Fourth, for hypertensive patients with comorbidities, the complexity of polypharmacy and potential drug-drug interactions represent an important consideration that procurement-level analyses cannot address; computational approaches for modeling interaction risks in multimorbid populations have been developed and could complement future pharmacoepidemiological studies (Wang et al., 2025). Fifth, incorporating pharmacovigilance data would enable assessment of whether safety concerns differentially affect the utilization of NVBP versus originator products. Finally, qualitative research exploring prescriber and patient perceptions of NVBP drugs in underdeveloped regions would inform targeted implementation strategies.

## 5 Conclusion

This study evaluated procurement-level changes for four antihypertensive drugs following the second NVBP batch in Guangxi, an economically disadvantaged and multi-ethnic region of southwestern China. The policy was associated with substantial decreases in procurement prices and DDDc, and with immediate increases in DDDs that were not sustained as a stable long-term trend. These findings suggest that while the NVBP policy achieved its primary price-reduction goal for these four drugs, further efforts may be needed to support consistent procurement volume patterns. Policy refinements could consider supply chain strengthening, alignment of procurement schedules with clinical demand cycles, and strategies to address stakeholder acceptance of NVBP products. The observed experience in Guangxi may offer preliminary insights for other regions or countries implementing centralized procurement in settings with limited healthcare resources, though any extrapolation beyond these four drugs and this region should be made with caution given the study’s methodological limitations. Future evaluations incorporating longer observation windows, control groups, patient-level data, and broader drug coverage are essential to fully understand the impact of NVBP policy.

## Data Availability

The original contributions presented in the study are included in the article/Supplementary Material, further inquiries can be directed to the corresponding authors.
