# Supplementary material for: Impact of the national centralized volume-based procurement policy on antihypertensive drug procurement, price, and volume in Guangxi: an interrupted time series analysis of procurement data
Source: Front Pharmacol. 2026 Jun 29;17:1810714. doi: 10.3389/fphar.2026.1810714 (PMC13357402; doi:10.3389/fphar.2026.1810714)
Supplement: Supplementary file 1 [file Table1.docx]

Table S1 Residual diagnostic tests for ln(DDDs) models.

| Drug | Shapiro‑Wilk *p* | Ljung‑Box (6) *p* | Breusch‑Pagan *p* | Seasonally adjusted |
| --- | --- | --- | --- | --- |
| Olmesartan | 0.478 | 0.087 | 0.957 | No |
| Candesartan Cilexetil | 0.928 | 0.081 | 0.979 | No |
| Terazosin | 0.966 | <0.001 | 0.510 | Yes |
| Indapamide | 0.284 | 0.048 | 0.253 | Yes |
