# Supplementary material for: Impact of the national centralized volume-based procurement policy on antihypertensive drug procurement, price, and volume in Guangxi: an interrupted time series analysis of procurement data
Source: Front Pharmacol. 2026 Jun 29;17:1810714. doi: 10.3389/fphar.2026.1810714 (PMC13357402; doi:10.3389/fphar.2026.1810714)
Supplement: Supplementary file 2 [file Table2.docx]

Table S2 Residual diagnostic tests for ln(DDDc) models.

| Drug | Shapiro‑Wilk *p* | Ljung‑Box (6) *p* | Breusch‑Pagan *p* | Seasonally adjusted |
| --- | --- | --- | --- | --- |
| Olmesartan | 0.107 | 0.008 | 0.001 | No |
| Candesartan | 0.007 | 0.638 | 0.001 | No |
| Terazosin | 0.020 | 0.382 | 0.002 | No |
| Indapamide | <0.001 | 0.430 | 0.001 | No |
